# Next-generation sequencing in chronic lymphocytic leukemia: recent findings and new horizons

**DOI:** 10.18632/oncotarget.19525

**Published:** 2017-07-24

**Authors:** Ana E. Rodríguez-Vicente, Vasilis Bikos, María Hernández-Sánchez, Jitka Malcikova, Jesús-María Hernández-Rivas, Sarka Pospisilova

**Affiliations:** ^1^ Department of Molecular and Clinical Pharmacology, University of Liverpool, Liverpool, United Kingdom; ^2^ IBSAL, IBMCC, Centro de Investigación del Cáncer, Universidad de Salamanca, CSIC, Hospital Universitario de Salamanca, Salamanca, Spain; ^3^ Central European Institute of Technology, Masaryk University, Brno, Czech Republic; ^4^ Department of Internal Medicine – Hematology and Oncology, Medical Faculty MU and University Hospital, Brno, Czech Republic; ^5^ Hematology Department, Hospital Universitario, Salamanca, Spain; ^6^ Department of Medicine, Universidad de Salamanca, Salamanca, Spain

**Keywords:** chronic lymphocytic leukemia, next-generation sequencing, clonal evolution, immunogenetics, CLL prognosis

## Abstract

The rapid progress in next-generation sequencing technologies has significantly contributed to our knowledge of the genetic events associated with the development, progression and treatment resistance of chronic lymphocytic leukemia patients. Together with the discovery of new driver mutations, next-generation sequencing has revealed an immense degree of both intra- and inter-tumor heterogeneity and enabled us to describe marked clonal evolution. Advances in immunogenetics may be implemented to detect minimal residual disease more sensitively and to track clonal B cell populations, their dynamics and molecular characteristics. The interpretation of these aspects is indispensable to thoroughly examine the genetic background of chronic lymphocytic leukemia. We review and discuss the recent results provided by the different next-generation sequencing techniques used in studying the chronic lymphocytic leukemia genome, as well as future perspectives in the methodologies and applications.

## The emergence of next-generation sequencing in the field of the chronic lymphocytic leukemia

Chronic Lymphocytic Leukemia (CLL) is a disease that displays extreme clinical heterogeneity [1]. This heterogeneity reflexes CLL’s marked molecular diversity [2], the analysis of which has led to the identification of a handful of biomarkers. Despite the significant progress in developing therapeutic options in CLL (extensively reviewed in [3, 4]), which has improved patient survival, CLL remains largely incurable and its course difficult to predict. Hence, the need to understand the disease’s heterogeneous features is essential in order to characterize each CLL case and eventually proceed to more tailor-made therapeutic approaches. The technological advancements emerging in the field of molecular analyses are providing the tools which will allow this to be done.

Next-generation sequencing technologies (NGS) have brought into play an unpreceded analytical depth to accommodate the characterization of the highly complicated genetic landscape of hematological cancers, with the case in point being CLL. Since the publication of the first complete cancer genome, obtained from a patient with acute myeloid leukemia [5], many groups have utilized NGS to further elucidate the biology of hematologic malignancies. These in turn have resulted in the assembly of large consortia such as the International Cancer Genome Consortium (ICGC) [6] and The Cancer Genome Atlas (TCGA) Research Network [7].

A glance back to the recent research on the elucidation of CLL genomics is enough to prove the usefulness of NGS technologies by virtue of the discovery of new drivers, including mutations in non-coding regions, and the elucidation of signaling pathways whose role was previously unknown or poorly understood [8–10].

Although CLL was originally considered a genetically stable disease, we now know that new genetic aberrations are acquired during clonal evolution and are characteristic of CLL development and relapse. NGS data have facilitated the detection of several tumoral subclones per sample and have illustrated different patterns of clonal evolution [2]. In addition, genetic loci can be sequenced in great depth and mutations existing at very low subclonal levels can be identified [11, 12]. The clones bearing these mutations can then be tracked during disease development and relapse after therapy.

Sequencing a specific genomic region in great depth also makes it possible to analyze the antibody repertoire more thoroughly, enabling a deeper understanding of the immune system with respect to the immunogenetic B cell features [13–15].

**Figure 1:**
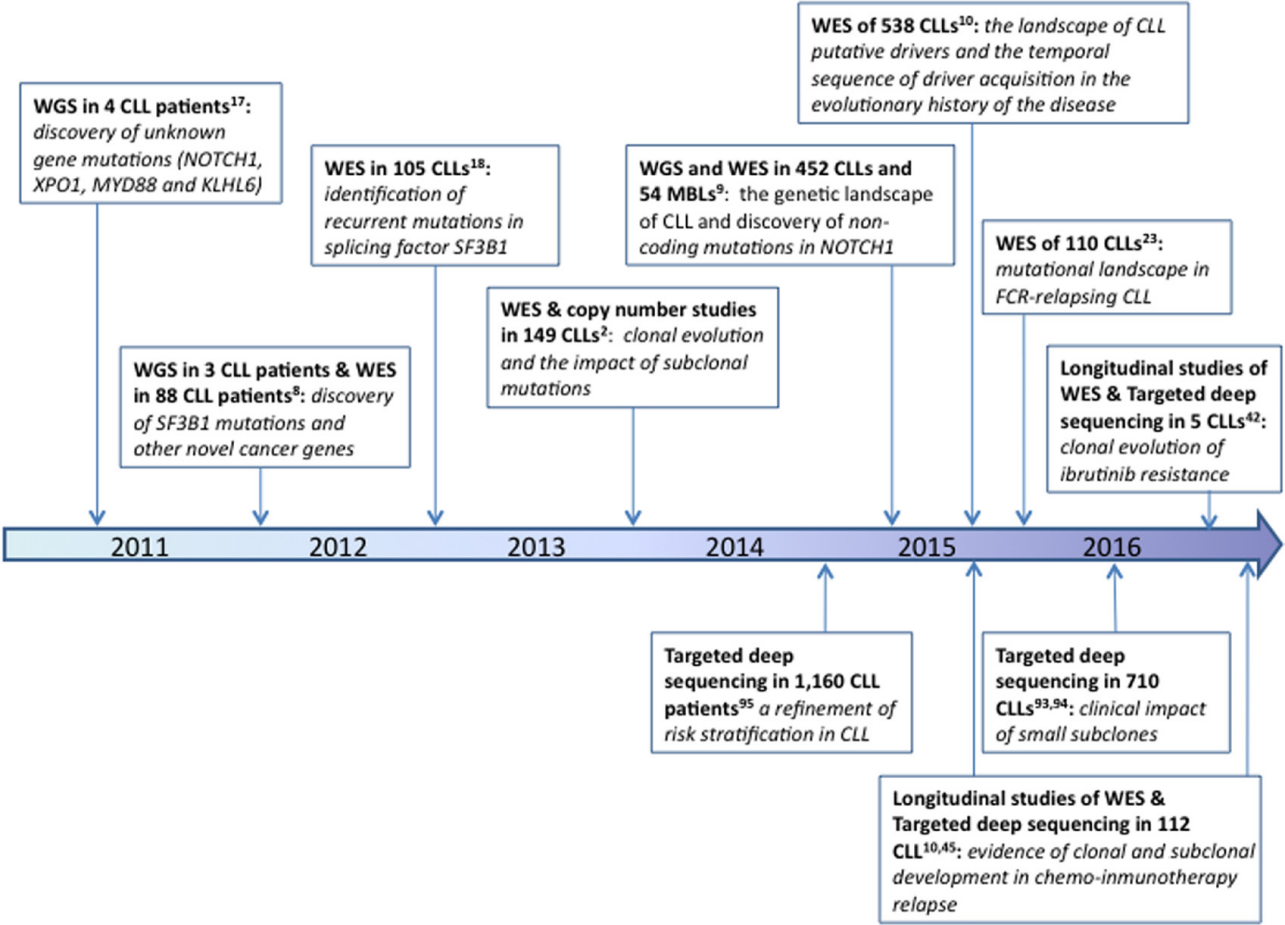
Timeline of the most relevant facts discovered in CLL by NGS studies

NGS allows us to explore the molecular pathways involved in disease pathogenesis and enables us to propose new genes which could be targeted for therapeutic purposes. Genes discovered using this approach may subsequently be included in prognostication gene panels, which in turn would ensure great coverage depth maximizing the results’ clinical utility. Figure 1 summarizes the most prominent discoveries in CLL in this “*NGS era”* and describes how the different experimental set-ups can assist in finding answers to various scientific questions.

Here, we review the recent insights of the various NGS studies in CLL, paying particular attention to the use of this method to give us a profound understanding of the disease and its clinical applications.

## Applications of NGS to the study of the CLL genome

**Figure 2:**
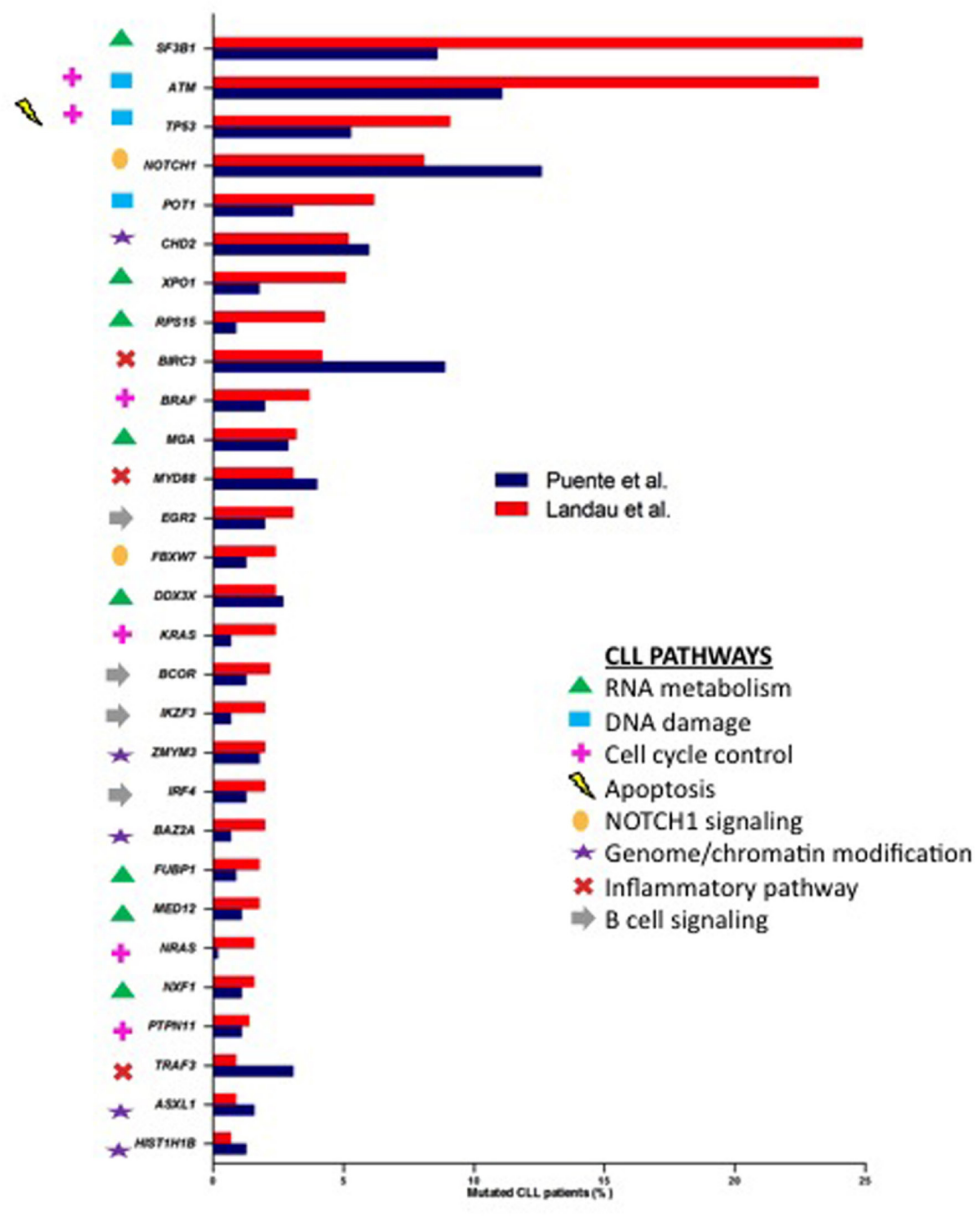
Percentages of samples affected by mutations in common CLL drivers from Puente *et al.* [9] (blue) and Landau *et al.* [10] (red) studies Genes were marked with different symbols according to the biological pathways involved.

Whole-genome sequencing (WGS) and whole-exome sequencing (WES) data are now available for extended patient series with more than 800 published CLL exomes or genomes [2, 8–10, 16–21], Landau *et al*. and Puente *et al.* are amongst the studies providing the most comprehensive exploration of CLL’s mutational landscape. These NGS approaches have revealed a vast genetic heterogeneity in CLL patients with a small number of genes mutated in approximately 10–15% of cases, and a large number of genes mutated at a lower frequency (< 10%) (intertumoral genetic heterogeneity). In parallel, as NGS enables the genome-wide detection of mutations between tumor cells, the genetic heterogeneity within malignant cells of the same patient (intratumoral heterogeneity) has also been accessed. Compared with solid tumors [22], CLL with a mutation rate of 0.60-0.87/Mb is a low genomic-complex disease with an average of 15.3-26.9 somatic mutations per patient, according to Landau *et al.* [10] and Puente *et al* [9], respectively. The early WGS/WES studies not only corroborated known CLL-associated alterations, such as somatic mutations in *TP53* and *ATM*, but also described a number of novel somatically mutated genes [8, 16, 17]. Amongst them, *NOTCH1* and *SF3B1* were identified as the most recurrently mutated genes with relatively higher frequencies than other candidates such as *MYD88*, *POT1*, *CHD2*, *XPO1*, *BIRC3*, *FBXW7* and *DDX3X*. Moreover, the most recent studies have increased the number of recurrently mutated genes, identifying previously unrecognized genes [9, 10]. It is noteworthy to mention some of them, such as *RPS15* [23], *EGR2* [24], *NFKBIE* [25] and *SETD2* [26]. The comparison between the two large cohort studies [9, 10] resulted in a significant overlap of 29 commonly mutated driver genes (Figure 2) albeit with existing discrepancies; the mutation frequencies were marked variable within common genes, and even some genes were exclusive of each study. These differences could be related to each cohort’s clinical characteristics: Puente *et al.* [9] analyzed only untreated CLL samples whereas over 6% of the patients were treated before the sequencing in Landau *et al* [10]. Another reason could be the different bioinformatics algorithms used in each study for variant calling as well as the definition of a mutated gene as a driver. It has been reported that the list of significantly mutated genes is different depending on the chosen computational method [27]. Incrementing the number of CLL cases analyzed by WGS or WES will identify even more significant mutated genes. Based on a saturation analysis and taking into account CLL background mutation rate, it has been estimated that an analysis of ∼2000 samples would be enough to confidently identify recurrent mutated genes present in 1–2% of CLL patients [28].

Whereas WES provides information about coding DNA regions, WGS allows extensive detection of all abnormalities including non-coding regions. The application of WGS has led to the discovery of the most frequent recurrent non-coding mutation located in the 3′ untranslated region of *NOTCH1* [9]. This splicing event is predicted to increase the stability of the NOTCH1 protein [9]. Furthermore, a small intergenic region of chromosome 9p13 was enriched for somatic mutations resulting in the reduced expression of the B-cell-specific transcription factor *PAX5* [9].

The large-scale comprehensive genetic characterization of CLL samples by WES and WGS studies has allowed us to better characterize the cell signaling pathways deregulated in CLL. Eight key cellular pathways (RNA metabolism, DNA damage, cell cycle control, apoptosis, NOTCH1 signaling, genome/chromatin structure, inflammatory pathway, B-cell signaling) have been described as recurrently mutated at different levels (Figure 2) [9, 10]. The emergence of NGS methodologies has also led to the discovery and extensive annotation of CLL driver genes implicated in previously unknown CLL-related pathways such as RNA metabolism (*SF3B1*, *XPO1*, *RPS15*, *MGA*, *DDX3X*, *FUBP1*, *MED12*, *NXF1*). After an explosive growth in our understanding of CLL genetics, now it is time to assess the biological impact of the somatic mutations and how they affect cellular fitness. In this line, the biological relevance of some of them, such as mutations in *POT1*, *CHD2*, *MED12*, *SAMHD1,* has been further explored by functional analyses [29–32].

A particular pathway might be further analyzed in detail by targeted resequencing using a panel of chosen genes. Such a study involving the mutational screening of 18 core complex genes within the NF-κB pathway was performed in a cohort containing 315 cases and in concordance with the previous study, *NFKBIE* was found to be the most frequently mutated gene. It was further shown that the mutations were associated with inferior outcome [25]. On the same line, targeted NGS was employed on 9 genes related to the p53 pathway in a cohort of 180 CLL patients [33], and the identified mutations’ impact on the transcriptional activation of p53 target genes was explored.

Genome-wide sequencing strategies have revealed novel candidate CLL driver genes within regions of copy number alterations. Apart from the known *TP53*/del(17p13) and *ATM*-*BIRC3*/del(11q22-23) associations, new regions have recently been identified, such as SETD2/del(3p21), NFKB2/del(10q24), MGA/del(15q15.1) [9, 10]. New structural variations have also been reported, particularly structural rearrangements on chromosome 1, 2, 13, and 14 [9, 20]. Surprisingly, a small subset of CLL had complex rearrangements such as chromothripsis and chromoplexy [9, 20, 34].

An additional benefit to applying NGS approaches to the study of large cohorts is the enhanced power to explore the relationships between driver lesions based on the patterns of their co-occurrence. This analysis revealed significant relationships between several alterations, including not only the already known high co-occurrence of *NOTCH1* mutations/chromosome 12 trisomy, but also the previously undescribed high co-occurrence between mutations in *BIRC3* and trisomy 12, mutations in *SF3B1* and *POT1*, as well as mutations in *NOTCH1* and *MGA*. Other alterations, such as del(13q) with trisomy 12, co-occur less frequently [9, 10]. While interactions between genetic alterations could synergistically act to enhance tumor growth, lack of co-occurrence could indicate that alterations have highly similar downstream effects, and hence would lack further evolutionary advantage to the tumor cell.

## NGS to study clonal evolution in CLL

Genome-wide sequencing studies have also shed light on CLL pathogenesis. CLL is preceded by a pre-malignant state known as B-cell lymphocytosis (MBL). MBL was shown to carry mutations in some CLL drivers, and the existence of clonal evolution was associated with a shorter progression time to CLL [35, 36]. MBL cases have a lower driver alteration burden than CLL patients, consistent with a model in which MBL-to-CLL evolution is accomplished by the progressive accumulation of driver alterations [9]. Regarding the origin of this disease, further studies by NGS are still necessary to clarify the cell of origin since few recent studies have suggested that CLL genetic alterations are present not only in B lymphocytes but also in hematopoietic progenitors [37, 38].

NGS studies have revealed the conspicuous presence of multiple genetically defined subpopulations that fuel diverse evolution patterns. Primarily, two common clonal evolution patterns have been observed in CLL patients: linear evolution, in which one dominant clone acquires driver events over time, and branched evolution, in which several tumoral subclones coexist and evolve over time [2, 19]. In this line, WGS showed different temporal repopulation patterns after therapy that deviate from a stable equilibrium among subpopulations, to marked shifts in which one minor subclone entirely replaced the dominant clone over time [19]. In a further investigation, WES data were analyzed using a computational approach to infer subclonal populations through integrating allelic fraction information about somatic mutations with local copy number and sample purity. This study demonstrated that genetic subclonal population features are linked to poorer clinical outcome, implying that an active evolutionary process is the underlying basis of an aggressive disease [2]. A temporal order of clonal and subclonal mutations corresponding to earlier and later events was suggested, in which the copy number variations such as 13q deletion and trisomy 12 were identified as consistently clonal early events while mutations in *ATM*, *BIRC3* or *TP53* were identified as subclonal later genetic alterations [2, 10]. Regarding biallelic inactivation of the *ATM*, a clonal del(11q) used to appear earlier than mutations in *ATM* which affects the remaining allele as a second hit [10].

The absence of an intervening therapy was largely associated with stable subclonal composition over time [39]. In contrast, chemotherapy exposure predominantly resulted in marked clonal evolution. WES data on matched samples collected at the time of first progression and relapse of fludarabine, cyclophosphamide, and rituximab (FCR) therapy have revealed large clonal shifts between pre-treatment and relapse samples in the majority of cases [10] and a recurrent mutation in *RPS15* in a large proportion of relapsed CLL cases [23]. Recently, WES was used to demonstrate that CLL patients with acquired resistance to ibrutinib therapy could not only have mutations in *BTK* at the binding site of ibrutinib or its immediate downstream partner PLCy2 [40] but also other potential alternative resistance mechanisms, such as del(8p) with additional driver mutations [41].

In order to monitor disease initiation, progression and relapse, it is important to define which clones are most biologically and clinically relevant. Targeted deep sequencing allows us to determine whether or not the driver mutations responsible for progression or relapse are already present in very small subclones at diagnosis. Longitudinal studies have already shown that the majority of driver mutations are already present in the initial stages, often as small subclones [10, 42–44]. Nevertheless, the clonal competition between the individual subclones carrying different mutations is a highly-complex process and mutations in the same gene can be selected in one patient and counter-selected in another [44]. The only consistent finding concerns *TP53*-mutated subclones, which were uniformly shown to increase upon relapse, suggesting a fitness advantage under therapeutic selective pressure [10, 43].

## Analysis of immunoglobulin genes using NGS

The importance of analyzing the molecular characteristics of the B cell receptor (BcR) in CLL patient management was shown by two seminal studies reporting that the immunoglobulin heavy chain (IGH) rearrangement’s mutational load can be used as a prognosticator, distinguishing patients with a mutated BcR (M-CLL) and a more indolent disease course from CLL patients bearing unmutated clonotypic BcRs (U-CLL) [45, 46]. Subsequent studies shed light onto other BcR characteristics in CLL, such as VH-CDR3 stereotypy [47–51] as well as intraclonal diversification in a number of them [52, 53]. The SHM burden is one of the strongest prognosticators, but the disease heterogeneity is still present among the M-CLL and U-CLL major groups. The compartmentalization of CLL patients into subsets of stereotypy is a step towards the creation of more homogeneous prognostication groups. In fact, different subsets of stereotypy not only differ in terms of demographics, specific clinical observations and disease course [54–56] but also markedly differ in the presence or absence of certain genetic lesions (chromosomal or recurrent gene mutations). Cases in point are the high incidence of *SF3B1* mutations in the poor prognostic subset #2, or the asymmetric distribution of *NOTCH1* mutations in poor prognostic subsets in contrast to the high incidence of *MYD88* mutations in patients belonging to subsets characterized as prognostically good [57–61]. The advancements that massive parallel sequencing technologies bring to the field can assist in studying these crucial characteristics but also to more sensitively depict the immunogenetic background, study clonal diversity and dynamics and monitor the disease course [62, 63].

Introducing high-throughput sequencing technologies can improve disease monitoring by means of minimal residual disease (MRD) detection. Sensitivity in MRD detection is a critical issue, since half of the patients who have undergone allogeneic stem cell transplantation (allo-HCT) experience disease reoccurrence [64, 65], albeit associated with being long-term disease-free when the examination is negative for one year after allo-HCT [66, 67]. Logan *et al.* [68] reported achieving MRD sensitivity detection equal to 1:100,000 molecules. They concluded that disease relapses in cases with MRD positivity where the malignant clone was present in more than 1:100,000 sequences, less than one year after-HCT. Boyd *et al.* [69] successfully identified all clonal populations from several samples, to a level of 1:10,000 copies, and recapitulated the oligoclonal pattern of a CLL case, which was shown to exist by means of conventional sequencing and capillary electrophoresis analysis. Recently, a study was published by the European Initiative in CLL [70], where the ClonoSEQ assay for MRD detection was compared with flow cytometry approaches and showed successful clone detection at the level of 1:10,000 copies.

NGS can also advance studies dealing with clonal diversity and intraclonal dynamics issues; Campbell *et al.* [71] attempted to track the evolution of the malignant clone by developing a bioinformatics algorithm to distinguish true somatic mutations from amplification and sequencing errors, based on the analysis of a non-polymorphic locus as a control, and reported unexpected intraclonal diversity not revealed by previous approaches. Gabriel *et al.* [72] attempted to recapitulate the immunogenetic profile in CLL patients. With a 1.6 × 10^–4^ detection sensitivity, they documented specific IG gene combinations with biased associations in CLL compared with healthy individuals and the intraclonal variation of the mutational burden.

A few studies dealt with the examination of multiple productive rearrangements in CLL. Kriangkum *et al.* [73] employed ImmunoSEQ, a multiplex PCR system to amplify CDR3 sequences using gDNA as a template in 26 CLL patients, to conclude that the allelic exclusion mechanism remained active in all cases examined, and that the presence of multiple clones is more frequent in M-CLL cases. Furthermore, they documented partner clones detected at a frequency greater than 5 × 10^9^ cells/L persisted over time despite treatment. More recently, Stamatopoulos *et al.* [74] employed the LymphoTrack IGH Somatic Hypermutation Assay Kit by Immunoscribe to show that the presence of multiple unrelated productive IGH rearrangements in CLL patients exceeds the so far estimated frequency, reaching a percentage of almost 25%. Moreover, based on the mutational status of these rearrangements, they categorized the patients into five different subgroups and claimed that this categorization improves disease stratification and patient management.

Finally, Blachly *et al.* [75] used next-generation RNA sequencing to compute the complete sequence of IG transcripts from unselected RNA-seq reads. With successful VH CDR3 region acquisition and the mutation status of the rearrangements along with a relative quantitation of oligoclonal samples they concluded that RNA-seq may simultaneously provide multidimensional data i.e. gene expression profile, mutation information and IGH Somatic hypermutation (SHM) status.

Sanger sequencing provided an essential image of the immunogenetic environment in CLL. The upgrade to the NGS era is still in its infancy; virtually all the studies so far are limited to analyzing only the heavy chain of the BcR. Although mutational status assessment and/or gene use in immunoglobulin light chain (*IGL*) rearrangement is not common practice, information about *IGH* and *IGL* associations could improve the research on CLL ontogeny, for example by strengthening the reliability of the functional analysis of antibody reactivity. Two individual groups attempted to fill that gap by combining single-cell PCR with next generation sequencing approaches to deep sequence pairs of IG heavy and light chains [76–79].

The 98% cut-off distinguishing M- and U-CLL was chosen arbitrarily, and borderline cases were always considered to be dealt with carefulness. NGS studies have documented the need for a more thorough classification [74], especially since there are suggestions for SHM analysis to be included in outline clinical tests [80]. This dictates the necessity to ensure a precise distinction between true somatic mutations and amplification/sequencing errors; the latter may generate artificial diversity and thus continuous efforts in building algorithms for error correction are taking place. However, such error corrections must be used with caution since they may inadvertently underestimate repertoire diversity by removing rare sequences [81]. A lab-bench advancement that may prove useful on this track, is the introduction of unique molecular identifiers (UMIs) in the sequenced fragments which will also assist a more detailed image of the clonal dynamics. To this end a methodology proposed by Khan *et al.* [82] and Turchaninova *et al.* [83] employed the introduction of UMIs at the reverse transcription level and applied asymmetrical sequencing on seven paired-end CLL libraries constructed from a specific number of cells/molecules in order for all molecules to be sequenced multiple times; they achieved full region sequencing with an estimated error rate of 1:10,000bp per UMI, since only molecules that were sequenced at least ×5 times were evaluated.

Other issues such as the low heterogeneity of IG sequences that can affect cluster formation and the biases introduced to PCR by the fact that some molecules are amplified more than others are currently under the spotlight. These issues and experimental set-ups to work around them are well-described and discussed by Georgiou *et al.* [84] At the same time the Euroclonality-NGS consortium has been established, with its primary objective being the development and standardization of NGS assays for clonality assessment, MRD analysis and repertoire analysis at the pre-analytical (wet-based) and post-analytical (bioinformatics) level.

## Clinical utilization of NGS in CLL

NGS techniques have provided a better knowledge of the genetic complexity underlying the clinical heterogeneity of CLL. Most findings indicate a prognostic role for some of the new gene mutations and emerging data suggest their role in predicting a therapeutic response and tracking disease relapse (reviewed in [85]) (Table 1). New genomic developments could improve patient outcome in two ways: (1) allowing for a more precise prognostic estimation and, (2) identifying novel therapeutic targets. For the use of prognostication in the clinical setting, targeted NGS is particularly suitable as it allows the concurrent evaluation of chosen genes, or parts of genes that are relevant to a given disease [86]. In addition, it is now possible to combine mutation analysis with copy-number analysis, thereby enabling the simultaneous detection of known copy-number aberrations. Several commercially available gene panels have been designed to detect either somatic or germline mutations in tumor-associated genes. As for CLL, there is currently only one commercially available CLL-specific gene panel. This amplicon-based panel allows single nucleotide as well as copy number variant analysis for the nine most prognostically relevant genes (Table 2). The efforts to design an optimal gene panel that could be widely used for CLL prognostication and, more importantly, treatment-effect prediction, are still on-going. So far, several groups have published results for studies employing a custom prognostic gene panel intended for clinical utilization (Table 2) [87–92]. Some of the recurrently mutated genes were common to all gene panels (*TP53, NOTCH1, SF3B1*), while others were found in one study only. The first study, reported by Jethwa *et al.* in 2013, used the designed NGS gene panel to study intratumor heterogeneity and clonal evolution, and documented the presence of convergent mutations [87]. Two other studies explored the suitability of NGS gene panels for diagnostic purposes [88, 89]. Guieze *et al.* [90] used their custom NGS gene panel to characterize relapsed/refractory CLL patients. They reported that concurrent gene mutations (“multiple-hit”) are frequent in patients with relapsed/refractory CLL and are associated with worse outcome. Rigolin *et al.* explored the co-occurrence of mutations and other clinical and biological characteristics in patients analyzed at diagnosis. They confirmed a negative impact of multi-hit profile and they reported association of mutation occurrence with adverse molecular and genetic findings including *IGVH* unmutated status, high-risk FISH results, and the presence of a complex karyotype [92]. Nadeu *et al.* used a gene panel to study the clinical impact and clonal evolution of subclonal mutations [91]. The role of small subclonal mutations is a timely question and the study by Nadeau *et al.* together with a report by Rasi *et al.* [93] consistently documented that only subclonal mutations in *TP53* and not *ATM*, *BIRC3*, *SF3B1* or *NOTCH1* genes are of prognostic importance, thus further confirming the clinical significance of the small *TP53*-defective subclones suggested by previous studies focusing only on *TP53* gene [12, 94]. It is therefore now discussed whether patients with small *TP53*-defective subclones should also be appointed for non-chemotherapeutic treatment. This consideration is extremely important with respect to novel targeted therapies that appear to dramatically improve the outcome of CLL patients with *TP53* defects. To elucidate this issue, a prospective study conducted on a uniformly treated patient series is desirable.

**Table 1:** Clinical relevance of the most important mutated genes in CLL patients studied by NGS

Gene mutation	Frequency	Association with	Prognosis	References
*TP53*	5.3–50%*	Del (17p13) UM-*IGHV*	Poor	2, 8, 17, 21, 23, 91, 93, 95
*ATM*	9–23.2%	Del (11q23) UM-*IGHV*	Poor	2, 8, 17, 23, 91, 93
*RPS15*	0.88–19.5%	UM-*IGHV*	Poor	9, 10, 21, 23, 45
*BIRC3*	4–15.5%	Del (11q22-q23) UM-*IGHV*	Poor	2, 16, 17, 21, 91, 93
*NOTCH1*	4–24.1%	Trisomy 12 UM-*IGHV*	Poor	11, 17, 18, 21, 23, 45, 91, 93
*SF3B1*	5–24.9%	Del (11q22-q23) ATM mutations UM-*IGHV* ZAP70 expression	Poor	2, 10, 17, 18, 21, 23, 45, 91, 93, 95
*MYD88*	1.5–10%	Del (13q14) M-*IGHV*	Good	2, 8, 10, 16–18, 45, 91, 95
*XPO1*	1.8–11.5%	UM-*IGHV*	Poor?	2, 10, 17, 18, 45, 91, 95
*FBXW7*	1.3–5%	Trisomy 12	Poor	2, 8, 10, 17, 45, 95
*SAMHD1*	2–9.8%	LOH on chromosome 20	Poor?	2, 10, 29, 45, 91

*At the time of diagnosis, *TP53* mutations are reported in approximately 5% of patients. On the other hand, in patients with relapsed and refractory CLL, the prevalence can be up to 50% of patients.

**Table 2:** Summary of CLL-specific prognostic gene panel designed by different studies

Study			Jethwa et al 2013^87^	Sutton et al 2015^88^	Vollbrecht et al 2015^89^	Guieze et al 2015^90^	Nadeu et al 2016^91^	Rigolin et al 2016^92^	Commercially available panel
Number of patients analyzed			178	188	136	114	406	200	
Library preparation method			Amplicon - multiplex PCR	HaloPlex (Agilent)	Amplicon - multiplex PCR	TruSeq Custom amplicon panel (Illumina)	Access-Array system, (Fluidigm)	Agilent HaloPlex Target Enrichment kit	Multiplex PCR (Multiplicom)
Sequencing platform			GS Junior 454 (Roche)	HiSeq (Illumina)	MiSeq (Illumina)	MiSeq (Illumina)	MiSeq (Illumina)	Ion Torrent PGM (Life technologies)	MiSeq (Illumina), Ion PGM (Life Technologies)
Limit of detection achieved			3%	10%	5%	8%, 30%*	0,3%	5%	Depending on samples per run analyzed
Total number of genes			9	9	15	9	5	20	9
Genes and regions covered	Genes included in more than one study	*ATM*	―	all coding exons	all coding exons	not specified	exons 2–63	exonic regions	all coding exons
		*BIRC3*	―	all coding exons	―	not specified	exons 2–9	exonic regions	all coding exons
		*MYD88*	exons 3, 5	all coding exons	all coding exons	not specified		exonic regions	all coding exons
		*NOTCH1*	exon 34	all coding exons	all coding exons	not specified	exons 26, 27, 34 and 3’UTR	exonic regions	all coding exons
		*PIK3CA*	exons 9, 20	―	exons 9–11, 20–21	―	―	exonic regions	―
		*SF3B1*	exons 14, 15	all coding exons	all coding exons	not specified	exons 14–16 and 18	exonic regions	all coding exons
		*TP53*	exons 4–10	all coding exons	all coding exons	not specified	exons 4–10	exonic regions	all coding exons
		*XPO1*	―	all coding exons	exons 12–13, 15,	not specified		exonic regions	all coding exons
	Other genes		*BRAF, EZH2, KRAS, NRAS* (selected gene regions)	*KLHL6*, *POT1* (all coding exons)	*BTK, CD79B, DDX3X, FBXW7, MAPK1, PIK3CD, PTEN, PTPN6* (selected gene regions)	*MED12, SAMDH1* (gene region not specified)	―	*BRAF, CDKN2A, PTEN, CDH2,* *DDX3X, FBXW7, KIT, KLHL6, KRAS, NRAS, POT1, ZMYM3 (*exonic regions)	FBXW7, *POT1* (all coding exons)

*Depending on the sequencing depth

Prospective multi-institution NGS studies of larger patient cohorts will allow us to integrate the newly discovered genetic lesions into a comprehensive prognostic model based on chromosomal abnormalities, gene mutations and also *IGHV* mutational status. This might improve patient prognostication and facilitate the introduction of testing into general practice. Efforts to devise an integrated prognostic model have already been made [95–97], although with partially discordant results, which may be related to differences in the design and analysis of the studies. Moreover, it is becoming evident that assessing the mutational complexity might be more informative than dissecting a specific prognosis for each driver as it was reported that cumulative number of driver mutations per tumor had a progressively worse effect [9, 90, 92].

Despite the still-widening list of prognostically significant genes, the only biomarker that influences the treatment strategy remains the presence of *TP53* defects [98], and to date, there has not been enough evidence to support the use of *NOTCH1*, *SF3B1*, *BIRC3* or *ATM* mutations, or CLL subclonal composition in making clinical decisions. Prospective trials with larger series of patients are needed to stratify CLL patients based on these mutations, in order to confirm their clinical impact and to address the possible use of these markers to adapt treatment.

Recent works have tried to recapitulate the biological consequences of some of the described mutations by grouping together genes belonging to the same biological pathways, as the genetic activation or silencing of a pathway can occur on multiple levels, with a relatively consistent effect on the output of the overall pathway [23, 25, 33, 99]. The presence of recurrent mutations affecting the activity of relevant cellular signaling pathways could indicate a key role for these mutations in CLL, and targeting these genes and pathways could provide effective therapy. Future directions should go into understanding the pathway map, which could lead to successful targets, as it can be witnessed currently in the clinical use of BCR signaling inhibitors [100]. Whilst there are currently no agents in CLL clinical trials to target *NOTCH1* and *SF3B1*, they are clearly promising targets and a number of agents targeting these genes and pathways are under development [101, 102]. *NOTCH1* is a well-established therapeutic target in T-cell acute lymphoblastic leukemia (T-ALL), while it is possible that splicesome inhibitors could have activity against *SF3B1* defects.

## Conclusions and future directions

NGS approaches have expanded our knowledge of genetic changes in CLL and leukemic clonal architecture, and this could have important consequences for prognosis and the optimized management of patients. CLL’s mutational profile is characterized by a relatively small number of somatic mutations per patient, a few recurrent mutations at moderate frequency, and a long list of recurrent low-frequency mutated genes. The abundance of these genomic aberrations in CLL has illustrated its huge biological heterogeneity. In fact, these genetic lesions are present not only in coding regions but also in non-coding regions. The advent of NGS has also allowed us to further refine the prognostic impact of *IGHV* rearrangement, enabling the identification of subclones with different *IGHV* rearrangements which would otherwise not be possible with Sanger sequencing, considerably less sensitive.

Technically, the NGS studies have proved the applicability, sensitivity and reproducibility of targeted sequencing approaches in CLL. However, several aspects need to be resolved before NGS can be implemented as part of routine diagnostics. Firstly, bioinformatics algorithms for data analysis represent an important obstacle. At present, multiple commercial as well as free software tools are available and many laboratories have developed their own in-house methodologies. Therefore, the inter-reproducibility of obtained results is an issue to be solved, particularly in the case of low-level subclonal mutations, and studies comparing the results obtained by different approaches are warranted and ongoing. Secondly, there is currently no agreement on the final list of genes to be included in the CLL-specific prognostication panel. The studies evaluating the prognostic impact of mutations in particular genes are to some extent contradictory, moreover, new potential driver mutations are continually being discovered and the gene selection may change with novel targeted therapies as the new mechanisms of resistance to these therapies have already been emerging.

The exploitation of NGS in prognostication and treatment prediction seems likely to become widespread in the near future; however, despite the rapid progress, significant work on the validation of new marker optimization and harmonizing the process between laboratories is still required before it can be used routinely in a clinical setting.
